# Recurrence and chronicity of bonding disorders diagnosed using the structured interview: case report

**DOI:** 10.3389/fpsyg.2024.1464417

**Published:** 2025-02-10

**Authors:** Yumi Nishikii, Yoshiko Suetsugu, Hiroshi Yamashita, Keiko Yoshida

**Affiliations:** ^1^Department of Pediatrics, National Hospital Organizations Nagasaki Hospital, Nagasaki, Japan; ^2^Department of Child Psychiatry, Kyushu University Hospital, Fukuoka, Japan; ^3^Department of Health Sciences, Kyushu University Faculty of Medicine, Graduate School of Medical Sciences, Fukuoka, Japan; ^4^Department of Medical Sciences, Kyushu University Faculty of Medicine, Graduate School of Medical Sciences, Fukuoka, Japan; ^5^Iris Psychiatric Clinic, Fukuoka, Japan

**Keywords:** bonding disorder, mother-infant relationship, perinatal mental health, maltreatment, neonatal health

## Abstract

**Introduction:**

Although emotional rejection, a core concept of bonding disorders, and pathological anger, which may harm the baby, can coexist, they have different clinical features and require different intervention strategies. Only limited reports have been published on the recurrence and chronicity of emotional rejection. To clarify this, in-depth investigations that utilize structured interviews rather than self-reported questionnaires are required.

**Methods:**

The participant was a 29-year-old woman at the first stage of delivery who had experienced three childbirths with different degrees of bonding disorders. We applied a section named “Mother-infant relationship” within the 6th Stafford interview developed by Brockington, which was used to assess bonding disorders, to report this case systematically. We also used the criteria for disorders of the mother-infant relationship developed alongside the interview.

**Results:**

Bonding disorders were diagnosed for this participant, with the first child as “threatened rejection” and the second and third as “mild disorders” (delayed positive feelings). Each improved with treatment within approximately 1 year; however, rejections recurred at different degrees when the next child was born. She was also diagnosed with pathological anger towards her first child, episodes of postpartum depression, and complaints of insomnia after the birth of all three children.

**Conclusion:**

Emotional rejection, pathological anger towards the baby, and infant-focused anxiety, in this case, should be diagnosed individually and appropriate care should be provided for each. Cases systematically documented using the Stafford Interview should be accumulated to facilitate clinical and research work on bonding disorders.

## 1 Introduction

Owing to research in the past two decades, after bonding disorders were recognized as a serious disorder in perinatal psychiatry in the 1990s, “bonding” is now considered as a positive emotional response towards the baby that occurs in the mother during the perinatal period. Therefore, this is distinguished from “attachment” which is the tie from baby to mother (Kinsey and Hupcey, 2013). Bonding involves finding the baby lovely and also feeling close to and irreplaceable with the baby and that this baby is her child, and is specific to each child, rather than the mother’s predisposing personality traits (Kumar, 1997).

Bonding disorder is a condition in which this bonding is impaired. Its core concept is emotional rejection (Brockington, 2016), which is a series of emotional responses that demonstrate the degree of impairment of the mother’s emotional proximity to her baby (i.e., lack of positive emotions, feeling like the baby is someone else’s, feelings of estrangement, desire to escape from the baby’s care, and rejection) (Brockington et al., 2006a). This disorder causes distress in childbearing women, affects parenting and mother-infant interactions, and leads to the infant’s mental health problems along with abuse and neglect. Psychiatrically, although bonding disorders can be associated with postpartum depression, they are also seen in mothers without depression. Furthermore, many mothers who are depressed have normal mother-and-infant relationships. The clinical severity, course, and treatment responsiveness of bonding disorders and depression or other psychiatric disorders are different. Therefore, considering bonding disorders as part of the clinical features of other psychiatric diseases is inappropriate. Accordingly, they are recognized as distinct in perinatal psychiatry (Brockington, 2016). In the Diagnostic and Statistical Manual, Fifth Edition, Text Revision (DSM-5-TR), bonding disorders fall under the “parent–child relational problem” as a “condition that may be the focus of clinical attention,” and is not considered as a mental disorder. Recent parent-brain studies extending knowledge related to mother–child relationships using neuroimaging or other neurobiological investigations have not included mothers exhibiting rejection.

Although no internationally standardized diagnostic criteria exist for bonding disorders, Brockington’s developed criteria can be used for mother-infant relationship disorders, which focus on emotional rejection. This also includes the mothers’ pathological anger toward their infants and infant-focused anxiety, which are significant to ensure the child’s safety. In recent psychometric research, self-report questionnaires, such as the Postpartum Bonding Questionnaire (PBQ) (Brockington et al., 2006b) and the Japanese version of the Mother-Infant Bonding Scale (MIBS-J) (Yoshida et al., 2012), have been used to assess bonding disorders; however, factor analysis cannot differentiate between anger and rejection (Brockington et al., 2006b; Kaneko and Honjo, 2014; Kitamura et al., 2015; Suetsugu et al., 2015; Yoshida et al., 2012). Therefore, individual interviews are the only way to identify feelings of anger and rejection. In addition, Brockington uniquely reported on women with recurrent bonding problems of different qualities at each of their children’s births (Brockington, 1996, 2018). We report the case of a woman who underwent three deliveries, each with a different degree of bonding disorder. Rather than narratively describing the case, we report the case based on the results of a structured interview and previously mentioned diagnostic criteria. The relationship between depression and bonding disorders that occurred after the first birth was reported in 2019 (Nishikii et al., 2019).

## 2 Case description

The 29-year-old Japanese woman grew up with middle-class parents in Japan and graduated from junior college. She had a history of eating disorders and anxiety in her teens. Her parents and relatives had no history of psychiatric disorders. She married a healthy man at the age of 29 years and lived with him without financial difficulties. Soon thereafter, she had her first unplanned pregnancy. Since her husband and her parents were pleased with the pregnancy, she welcomed it. However, she was indifferent to her fetus owing to her unreadiness of childbearing and fear of the forthcoming delivery. She gave birth to a healthy full-term baby girl. After discharge from the maternity hospital on the fifth postpartum day, she stayed at her parents’ home and raised her baby with the baby’s grandparents’ support. She was anxious regarding her infant’s health and growth. Two weeks postpartum, she experienced depressive mood and insomnia. She felt especially distressed about her baby crying at night. Subsequently, she was referred to our clinic by the midwives and visited 1 month postpartum. She was diagnosed with perinatal depression and prescribed sertraline. After her depression improved with medication, she was often frustrated with the baby. Although she left her parent’s home and started living with her husband and her baby 5 months postpartum, she still required her mother’s help for babysitting long after that.

Returning to a woman’s parents’ home for a few months to give birth, usually 1 month before delivery and a couple of months after giving birth, is a culturally accepted traditional Japanese perinatal care style. However, she required her mother’s baby care support for a longer period than usual. She finally recovered from depression and discontinued her medication 1 year postpartum.

One year after the first birth, she became pregnant with her second child. However, despite the planned pregnancy, she regretted it when she discovered it. She had severe hyperemesis gravidarum, a depressive mood, and no affection for her fetus. She delivered a healthy, full-term baby girl. After birth, the mother experienced insomnia, anxiety, and depression. On the fourth postpartum day, she revisited our clinic and was diagnosed with depression once more, which was milder than the first time. She was treated with sertraline for a year. She stayed at her parents’ home for baby care and support for 5 months. Although she was frustrated by the second baby’s crying, the baby was easy to soothe. Meanwhile, she was often angry at the older child. After she returned home, she cared for the second child, while the grandmother and her husband cared for the first child and helped with other housework.

One year after the second birth, she discovered that she had an unplanned third pregnancy. Although she was unhappy about the pregnancy, she refused to get an abortion recommended by her husband. The couple agreed to continue with the pregnancy. She gave birth to a healthy boy and stayed with her three children at her parent’s home for 6 months. Her depression re-occurred second week postpartum, and she reduced her childcare burden earlier by discontinuing breastfeeding and switching to formula. She was treated with sertraline for 2 years for depression, irritability, and insomnia caused by the stress of raising three children.

## 3 Assessments

### 3.1 General assessment

We conducted a clinical psychiatric interview with this woman and made a diagnosis using the DSM-5. Her three children were each assessed during this study period using a standard mental health screening tool of Japanese public health, which included assessments for physical growth, feeding/eating, sleeping regulation, motor activity, emotional expression, social communication and interaction, language and cognition.

### 3.2 Diagnostic instruments for bonding disorders

#### 3.2.1 The 6th edition of the Stafford interview

The 6th edition of the Stafford Interview was used to diagnose this woman’s emotional disturbance towards her babies in detail (Brockington et al., 2017). This structured interview was developed by Brockington (1996) to explore pregnancy-related psychiatric disorders and underwent its 6th revision in 2014. Brockington permitted Yoshida (one of the authors of this paper) to translate this interview into Japanese in 2015, and the Japanese version was published in 2019. In this case, we applied a section of the interview named the “mother-infant relationship” which was used as a self-standing instrument to assess bonding disorders. According to the interview instructions, trained professionals YN (or YS) and KY conducted the interviews and coded the mother’s responses for each child. Consensus on the ratings was reached by adding a third rater (YS or HY) and discussing the mothers’ responses.

#### 3.2.2 Criteria for disorders of the mother-infant relationship

This diagnostic classification was developed to categorize participants in a 2006 study that explored bonding disorders in 205 mothers (Brockington et al., 2006b). It divides emotional rejection into two groups: severe disorder is “established rejection, “and rejection below the diagnostic threshold is “threatened rejection. “Under the threshold of rejection but with milder problems, delayed or secondary loss of positive feelings towards the baby are classified as mild disorders.

Additional diagnoses include “pathological anger, “which could cause harm to the baby, and “infant-focused anxiety, “which is avoiding the baby owing to anxiety regarding the baby’s care.

## 4 Results

### 4.1 Results of the general assessment of the mother and her children

This woman was diagnosed with perinatal depression via the DSM-5 at each of the three perinatal periods. Among her three children, none had physical growth problems. The three children were 6, 4, and 2 years old, respectively, at the end of the study and had no problems with physical growth, motor, language and cognitive development. The first child was optimistic, even when her mother was frustrated. The second child was somewhat nervous and behaved sensitively to her mother’s attitude. The third child tended to cling to his mother.

### 4.2 Results from the Stafford interview

**Figure 1:**
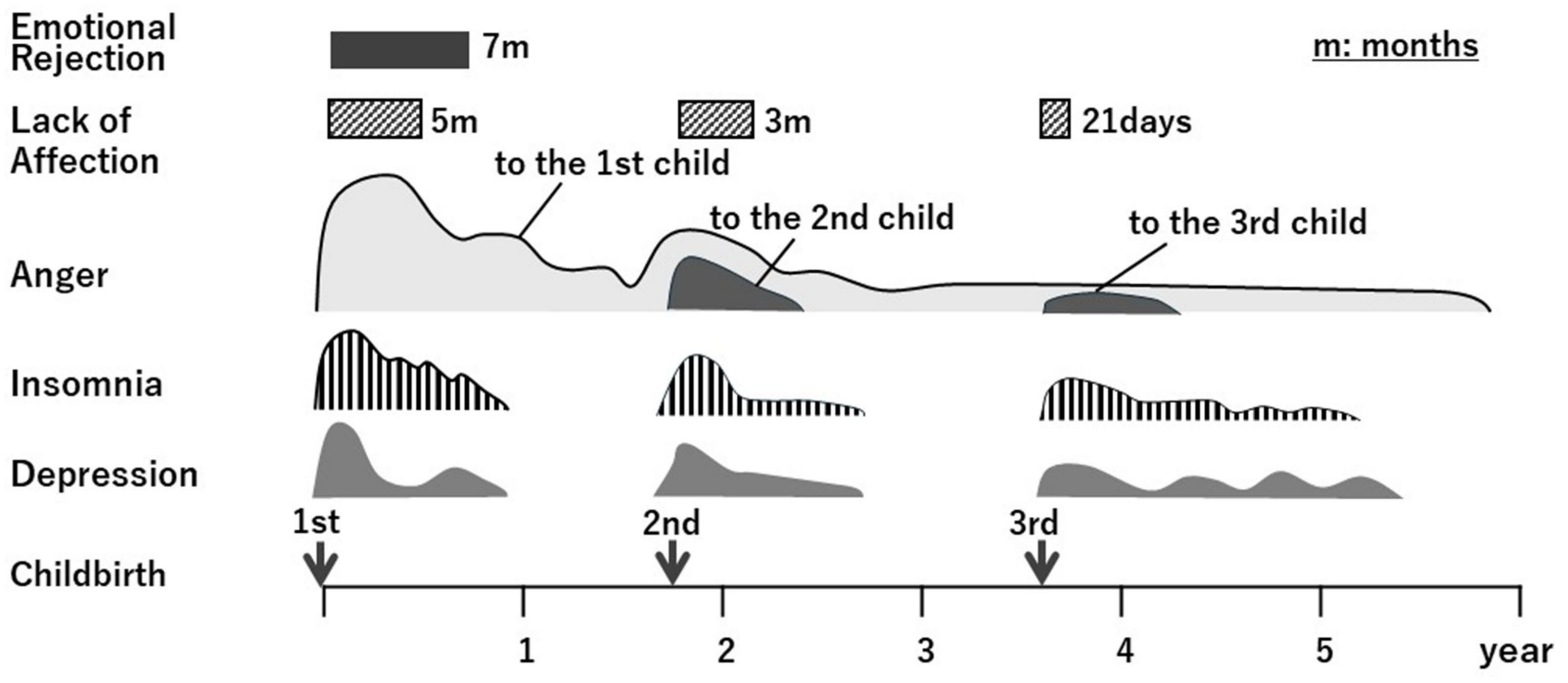
The clinical course of this case’s bonding problems toward her baby.

**Figure 2:**
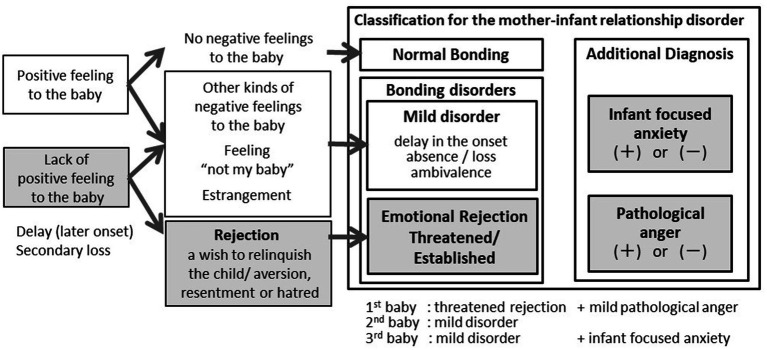
Different bonding disorders of this case in every three children on the diagnostic flow chart.

Table 1 presents the results of the Stafford interviews regarding the mother’s feelings towards each of the three children individually. Figure 1 illustrates the course of the emergence of symptoms of the mother-infant relationship disorder, along with depression and insomnia. Finally, we demonstrated the diagnostic classification for bonding disorders (Figure 2). Below, we summarize the narrative evidence of the mother’s feelings towards her children for each item in the Stafford interview.

**Table 1:** Results of the section on “mother-infant relationship” within the Stafford Interview.

No.	Items of the Stafford interview	For the 1st baby	For the 2nd baby	For the 3rd baby
Infant characteristics and maternal involvement in care				
158	Baby’s temperament	Cried but can be pacified (2)	Easily soothed (0)	Easily soothed (0)
159	Baby’s other problems	Constipation (1)	No problems (0)	No problems (0)
160	Infant development	Normal (1)	Normal (1)	Normal (1)
161	Mother’s involvement in infant care	Transferred the baby’s care to her mother (2)	Did baby care with her mother’s help (2)	Did baby care with her mother’s help (2)
162	Mother’s emotional over-involvement	No over-involvement (0)	No over-involvement (0)	No over-involvement (0)
163	Quality of emotional involvement	No enjoyment until 5 months	No enjoyment until 4 months	No enjoyment until 22 days
the mother’s emotional response to her infant				
164	Timing of positive feelings for baby	5 months after birth	3 months after birth	3 weeks after birth
165	Feeling of estrangement	Felt the baby was not her own (1)	No feeling of estrangement (0)	No feeling of estrangement (0)
166	Nature and strength of feelings for infant (historic)	Anger and rejection (4)	No positive feelings until 3 months (3)	Wished the baby was not like this (3)
167	Nature and strength of feelings for infant (present)	Ambivalent (2)	Felt beautiful but irritated when the baby cried (2)	Felt beautiful but irritated the baby following and crying (2)
168	Ideas of transferring care or escaping from maternal duties	Temporary transfer (4 at her worse)	No thoughts to transfer (0)	No thoughts to transfer (0)
169	Fantasies of infant loss	No such ideas (0)	No such ideas (0)	No such ideas (0)
Anger and abuse				
170	Angry response to infant	Stamped her foot bedsides (4)	Controlling anger by leaving the baby (3)	Irritated, but only felt internally (1)
171	Frequency of maternal anger	Most of the time (3)	often (2)	Most of the time (3)
172	Coping with maternal anger	Leave the baby crying (2)	Leave the baby crying (2)	No need for coping (0)
173	Child abuse	Abuse has not occurred (0)	Abuse has not occurred (0)	Abuse has not occurred (0)
174	Child neglect	No neglect (0)	No neglect (0)	No neglect (0)
175	Filicidal impulses and activity	No such ideas (0)	No such ideas (0)	No such ideas (0)

Rating codes in the Stafford interview instructions are shown in brackets.

### 4.3 Maternal narratives from the Stafford interview

#### 4.3.1 Infant characteristics and maternal involvement in care

This part of the interview provided evidence regarding the mother’s perceptions and concerns regarding the baby’s characteristics and mother-infant interactions.

The first child would stop crying when soothed but cried for hours at night. She suffered from insomnia owing to night-time crying and left the baby care to the grandmother. The mother did not enjoy caring for the baby until 5 months postpartum.

However, she could soothe the second baby easily, but at approximately 18 days of age, the baby cried at night, distressing her. However, the mother performed most of the childcare with her mother’s assistance. She had no desire to escape childcare for the second child. However, she could not provide affectionate parenting until the baby was 4 months old. The third baby was easiest to handle and soothe when held. The mother did most of the baby’s care and only asked her mother for help when she felt tired.

#### 4.3.2 Mother’s emotional response to her infant

This part of the interview revealed the timing of the emergence of positive feelings towards the baby, emotional rejection, and presence of other negative feelings.

After the first birth, the mother felt that the first baby was like a stranger. She regretted giving birth. The desire to escape from the baby’s care was strongest 3 days after birth and subsequently continued. She wanted someone to temporarily care for the baby for the first 3 months postpartum. She did not experience positive feelings towards the baby until 5 months postpartum. At 7 months postpartum, she still wanted to run away from caring for the baby when she was tired.

After the second birth, she found it difficult to cuddle the second baby and felt disappointed by her poor motherhood. Around 3 months postpartum, the baby started to laugh aloud, and she began to think it was beautiful, but she was irritated with the baby when it was crying in the evening.

After the third birth, the mother experienced a slightly manic mood but soon developed physical fatigue, depression, and anxiety and no longer felt that she had delivered the third baby. On the 22nd postpartum day, she finally thought that the baby was beautiful, but simultaneously, she was anxious when she was with him. Nine months later, he always followed his mother, calling her as soon as she left him. She thought, “Why does this baby not play alone? And I wish he was not like this.” She generally had positive feelings towards her third child.

#### 4.3.3 Anger and abuse

This part of the interview clarified pathological anger, how to control it, and acting out due to the loss of control over anger.

Transient bursts of intense anger towards the first baby, especially when the baby was crying and did not sleep, were felt immediately after birth and continued subsequently, but without impulses to hurt the baby. Several weeks after birth, the mother’s anger towards the baby peaked, and she was unable to control her anger, shouting at her baby and repeatedly stamping her feet on the ground beside the bed. The only way to control her anger was to leave the baby with the grandmother. At 7 months postpartum, she told herself to control her anger, and she sometimes left the baby crying.

The second baby frustrated the mother only when she cried at night. While the baby’s crying was intense in the evening, which lasted until approximately 7 months postpartum, she could control her anger and felt that it was easier to manage the baby. She was irritated until approximately 9 months after the birth of her second child, but most of her anger was directed towards her first child. Regarding the third baby, the mother felt angry when he followed her, but she could calm her anger naturally.

### 4.4 Diagnostic classification for bonding disorders

Figure 2 illustrates Brockington’s classification of bonding disorders and the mother’s diagnosis for each child (Figure 2). After the birth of all three children, the mother lacked positive feelings towards her children for 5 months, 3 months, and 22 days, respectively. Furthermore, she expressed a desire to escape the first baby’s care, not permanently, and left it to the grandmother, which led to the diagnosis of threatened rejection. Since she also screamed at the baby angrily and stamped her feet, her additional diagnosis was mild pathological anger. Since the mother did not desire to escape the baby’s care for the second and third births, she was diagnosed with a mild disorder. Although she was irritated towards the baby, she only experienced it in her mind and did not meet the criteria for pathological anger. For the third birth, because the mother reported fear regarding the third baby when she was with him but did not avoid contact, the additional diagnosis was mild infant-focused anxiety.

## 5 Treatments and outcomes

We first prescribed sertraline for postpartum depression for 1 year after the first and second births, which was effective. The third postpartum period was treated for 2 years owing to prolonged depressive symptoms due to the distress of raising three children and marital problems.

The best treatment for her insomnia was for the family to take on the responsibility of baby care at night, along with sleeping medication, as required. We provided individual psychotherapy to the patient along with education for family members regarding baby care support after the birth of the first and second children. One year after the birth of the third child, the woman required psychotherapy with a focus on marital relationships.

At the end of the study, she still felt occasional anger towards her first child but had coping skills and controlled her anger. She reported a feeling of bond with her three children.

## 6 Discussion

This case involves three childbirths with bonding disorders of various qualities. We systematically summarized the course of this case using structured interviews and diagnostic criteria to evaluate the bonding disorders.

The mother was diagnosed with emotional rejection as experiencing “threatened rejection” with the first child and “mild disorders” (delayed positive feelings) with the second and third. Although each rejection recovered within approximately 1 year, it recurred to different degrees when the next child was born. In the Brockington case series, four cases of recurrence with varying degrees of rejection were reported in multiple children (Brockington, 2018). The most severe rejection did not always occur in the first child. Details of the background factors that explain this variability in the degree of rejection remain unknown. Furthermore, while two cases in the case series with rejection recovered spontaneously, the other two exhibited chronicity in one child aged over 3 to 4 years in the absence of treatment. Therefore, recognizing that emotional rejection is repetitive and chronic along with a management plan for a safe and secure life for both mother and child is essential.

Coexisting depression and other psychiatric symptoms, along with bonding disorders, affect each other. In this case, the woman also presented with depressive symptoms during the three postpartum periods. Life events of pregnancy and childbirth, emotional rejection, burden of baby care, and sleep deprivation after birth lead to long-term stress for the mother and contribute to the development of depression.

The patient was diagnosed with severe insomnia and postpartum irritability. Brockington reported that after birth, insomnia, irritability, and depression were associated with pathological anger (Brockington, 2018). Pathological anger may also be present even in the absence of rejection and may be dangerous to the baby. Therefore, in addition to treating rejection, insomnia, irritability, and depression, these must be adequately addressed postpartum.

Based on the above diagnostic features, the treatment principles proposed by Brockington for rejection are: 1. deciding whether to raise the child with family; 2. treatment of comorbid depression; 3. reduction in the mother’s baby care burden; 4. helping the mother interact with the child; and 5. protecting the mother and infant’s safety (Brockington, 2018). In this case, help from the husband and woman’s mother played a significant therapeutic role in reducing the burden of baby care and assisting in mother-infant interaction.

This case report is valuable in demonstrating the differences in the long-term clinical course of emotional rejection and pathological anger and clarifying the diagnostic significance of these bonding problems. However, this study has some limitations. Our results were based on only the postpartum parts of interviews on mother and infant relationships. Hence, we could not clarify mothers’ rejection and anger risk factors. To understand the comprehensive aspects of bonding disorders, assessment during pregnancy and accumulation of the results using this interview’s demographic and antenatal sections is prudent.

This study offers insights into emotional rejection’s impact on perinatal women’s mental health and parenting. Our results suggest the importance of adequate care provided to mothers with rejection and have the potential to enable breakthroughs for perinatal mental health care in the future.

## 7 Conclusion

We reported the recurrence of emotional rejection with different severity levels for each child. We also found that pathological anger occurred with a different psychiatric background and clinical course from rejection. The Stafford Interview method helped clarify these points.

## Data Availability

The original contributions presented in the study are included in the article/supplementary material, further inquiries can be directed to the corresponding author.
